# Targeting Homologous Recombination in Notch-Driven *C*. *elegans* Stem Cell and Human Tumors

**DOI:** 10.1371/journal.pone.0127862

**Published:** 2015-06-29

**Authors:** Xinzhu Deng, David Michaelson, Jason Tchieu, Jin Cheng, Diana Rothenstein, Regina Feldman, Sang-gyu Lee, John Fuller, Adriana Haimovitz-Friedman, Lorenz Studer, Simon Powell, Zvi Fuks, E. Jane Albert Hubbard, Richard Kolesnick

**Affiliations:** 1 Laboratory of Signal Transduction, Memorial Sloan Kettering Cancer Center, New York, New York, United States of America; 2 Department of Cell Biology, Helen L. and Martin S. Kimmel Center for Stem Cell Biology, Skirball Institute of Biomolecular Medicine, New York University School of Medicine, New York, New York, United States of America; 3 Center for Stem Cell Biology, Memorial Sloan Kettering Cancer Center, New York, New York, United States of America; 4 Department of Radiation Oncology, Memorial Sloan Kettering Cancer Center, New York, New York, United States of America; East Carolina University, UNITED STATES

## Abstract

Mammalian NOTCH1-4 receptors are all associated with human malignancy, although exact roles remain enigmatic. Here we employ *glp-1(ar202)*, a temperature-sensitive gain-of-function *C*. *elegans* NOTCH mutant, to delineate NOTCH-driven tumor responses to radiotherapy. At ≤20°C, *glp-1(ar202)* is wild-type, whereas at 25°C it forms a germline stem cell⁄progenitor cell tumor reminiscent of human cancer. We identify a NOTCH tumor phenotype in which all tumor cells traffic rapidly to G2⁄M post-irradiation, attempt to repair DNA strand breaks exclusively via homology-driven repair, and when this fails die by mitotic death. Homology-driven repair inactivation is dramatically radiosensitizing. We show that these concepts translate directly to human cancer models.

## Introduction

Notch is a single-pass transmembrane glycoprotein receptor that plays key roles in lineage specification and differentiation processes during development, and in maintenance of stem cells in adult life [1]. Mammals have four Notch receptors, Notch-1 to Notch-4. A large literature supports a role for dysregulated Notch signaling in human malignancy. Constitutive Notch signaling is associated with over 50% of human T cell acute lymphoblastic leukemias (T-ALLs), which have activating Notch-1 mutations that drive tumorigenesis [2–4]. Elevated Notch activation is also implicated, directly or indirectly, in the pathogenicity of a variety of solid tumors, including breast, colorectal and pancreatic cancer [5]. Furthermore, a substantive literature implicates Notch in tumor progression and maintenance, in addition to tumor initiation. Notch signaling also leads to tumor cell resistance to conventional drug and radiation therapies [5–7]. In some contexts, however, NOTCH receptors are not tumorigenic, but rather act as tumor suppressors [8]. Such complexity underscores the need to carefully consider strategies to intervene in human NOTCH receptor signaling for therapeutic benefit. The current studies use *C*. *elegans* genetics to investigate potential pharmacologic approaches to NOTCH.

*C*. *elegans* contains two Notch family receptors, LIN-12 and GLP-1 [9]. LIN-12⁄Notch signaling plays roles in somatic tissue development such as in vulval precursor cell specification [10,11], while GLP-1⁄Notch signaling is a major regulator of germline development [12]. GLP-1 is expressed on the surface of a population of germline stem⁄progenitor cells (GSCs) in the distal *C*. *elegans* gonad, and is activated by binding Delta⁄serrate⁄LAG-2 (DSL)-family ligands produced by a single niche cell, the distal tip cell (DTC) [12,13] GLP-1 signaling promotes a proliferative germ cell state, and prevents germ cells from undergoing precocious meiosis. Thus, loss of GLP-1 signaling results in a severe proliferation defect and early meiotic entry [12], while constitutive activity yields a germline tumor with all germ cells remaining undifferentiated [14]. The expanding tumor eventually perforates the gonad, resulting in invasion of germ cells throughout the worm body, and early animal death [14,15]. Since Notch signaling normally maintains a population of self-renewing cells in the distal *C*. *elegans* gonad, the GLP-1 germ line tumor is considered to represent a stem cell tumor model [16–18].

At the molecular level, there is considerable similarity between human and *C*. *elegans* gain-of-function (*gf*) tumor-driving mutations. Here, we used *glp-1(ar202)*, a temperature-sensitive gain-of-function (*gf*) *C*. *elegans* mutant [19] to investigate Notch-driven tumor responses to radiotherapy. Similar to activating mutations in Notch1 that are associated with human tumors, this allele modifies the Notch extracellular negative regulatory domain [1] and leads to hyperactive Notch signaling [19]. We reasoned that this simple model would allow for detailed analysis of the fundamentals of the tumor response of the *C*. *elegans* “patient” to radiotherapy, hopefully providing insight that might be useful in designing mechanism-based approaches to Notch-driven human tumors.

A basic tenet of radiobiology posits tumor stem cell radiosensitivity is a critical determinant affecting radiocurability [20] with depletion of the stem cell compartment required for tumor cure. Mammalian cell lethality occurs predominantly via the reproductive (also known as mitosis-associated or clonogenic) cell death pathway, triggered by radiation-induced DNA double strand breaks (DSBs) [21–23]. DSB repair occurs mainly *via* the error prone non-homologous end joining (NHEJ) or the error free homology-directed repair (HDR) pathway [24], promoting tumor cell survival. Residual unrepaired or misrepaired DSBs, however, confer genomic instability [25], propagating chromosomal aberrations during post radiation mitotic cycles, eventually resulting in lethal chromatid⁄chromosomal translocations and recombinations, and reproductive demise of progeny [21,22]. While this concept implies the genetic blueprint of the DSB repair machinery determines inherent cell-specific radiosensitivity, the relative contribution of NHEJ versus HDR dysfunction to stem cell radiation lethality remains an issue of debate [23]. Here, we define for the first time a Notch-specific radiation response phenotype that allows for development of radiosensitizing strategies in *C*. *elegans* stem cell tumors. Further, we report that principles derived from this model translate directly to treatment of human T-cell lymphoblastic lymphoma CUTLL-1 tumor xenografts in mice, a classic pre-clinical model of human Notch-driven cancer [26].

## Materials and Methods

### Nematode strains

Wild-type N2, *glp-4(bn2)* and *ced-3(n717)* were provided by the Caenorhabditis Genetics Center (University of Minnesota). Strains were maintained as per Brenner [27] at 15°C. To study germ cell tumor, L4 larvae of *glp-1*(*ar202*)(GC833) were shifted to 25°C and progeny were collected at the stage indicated. Double mutant *glp-1(ar202);ced-3(n717)* worms were generated using single-worm polymerase chain reaction (PCR) as per [28]. The *ced-3* point mutation was confirmed in *glp-1(ar202);ced-3(n717)* by DNA sequencing. Primers for genotyping mutant are: *ced-3(n717)*: 5'-cggcttctttctccacacttgtta- 3' ⁄ 5'-ggcgcacaccccatttgcattg- 3' and for wild type *ced-3*: 5'—cggcttctttctccacacttgcta—3' ⁄ 5'-ggcgcacaccccatttgcattg- 3'; primers for genotyping mutant *glp-1(ar202)*: 5’ tttggagaatggtctttcct 3’ ⁄ 5’ gtcatgcaaatacaatccgtg 3’ and for wild type *glp-1*: 5’ tttggagaatggtctttccc 3’ ⁄ 5’ gtcatgcaaatacaatccgtg 3’.

### Worm RNAi by feeding

RNAi was performed essentially as per [29]. Single colonies of HT115 bacteria containing L4440 plasmids with cloned fragments corresponding to target genes were from Vidal and Ahringer RNAi feeding libraries. Each RNAi reagent was verified by DNA sequencing. Young adult hermaphrodites were placed onto NGM plates seeded with dsRNA-expressing or empty vector control bacteria (RNAi feeding plate). After overnight incubation, worms were transferred to an identical fresh RNAi feeding plate and allowed to lay eggs for 2h. RNAi phenotypes of synchronized F1 progeny were examined at the indicated times post radiation.

### Quantitative PCR

Worms were collected in Trizol reagent (Invitrogen) and subjected to three rounds of freeze cracking by alternating between liquid nitrogen and room temperature. Crude RNA extracts were collected and purified with RNeasy Mini Kit (Qiagen) according to manufacturer's instructions. 1 μg of total RNA was reverse-transcribed in 20 μl using the Thermoscript RT-PCR system (Invitrogen) at 50°C for 1h. Quantitative PCR was performed on the Applied Biosystems 7500 FAST Real Time PCR instrument with Taqman Gene Expression assay system. The IDs of *C*. *elegans* gene expression assay are: *mre-11*—Ce02480998_g1; *rad-51*—Ce02458920_g1; *atl-1*—Ce02479867_g1; *mus-101*—Ce02413322_g1; *cku-80*—Ce02445546_g1; *lig-4*—Ce02449042_g1; *hsr-9*—Ce02412427_g1; *rad-50*—Ce02482582_g1; *mec-7—*Ce02497588_g1. Expression level of each sample was standardized to *C*. *elegans mec-7* endogenous control standard. Knockdown was calculated as percentage remaining gene expression normalized to relevant non-silenced control.

### Germ cell quantification

Worms were fixed in ethanol and stained with DAPI using Vectashield (Vector Laboratories Inc.). Z-stack images were acquired with a 20x water objective at 2-μm intervals using a Leica Confocal Microscope. To quantify *C*. *elegans* germ cell nuclear numbers each entire z-stack was loaded into Volocity (version 5.3.1) as a single lei file. Then the entire area of visible DAPI- stained germ cells in one gonad arm was selected for analysis. If the two gonads were uneven size, germ cells from both gonads were measured and averaged. In the selected gonadal area threshold intensity was set high enough such that the program identified individual cells and excluded spaces between cells. The Volocity Program requires an approximate size guide to find objects. We determined the approximate nuclear volume experimentally by measuring volume from high magnification images of DAPI stained nuclei (63x, zoom 5). At least 100 nuclei from 4–5 worms per condition were measured. Volocity quantification was verified by hand counting of ~20 gonads from *glp-1(ar202)*. Generally, Volocity numbers were lower than hand counts, but differed by <5%.

### *glp-1(ar202)* tumor cell cycle arrest

Adult worms, raised at 15°C, were transferred to 25°C and allowed to lay eggs for 1.5h. After hatching, mid-L4 progeny were transferred to fresh plates and either irradiated at 480Gy, requiring 4h, and allowed to recover for 8h, or not. Relative germline nuclear DNA quantity was determined as per [30] with the following modifications: worms were stained with DAPI (Vector Laboratories Inc.) and all nuclei were quantified from position of cell diameter (CD) 6 through CD 15 from the distal tip, or CD -1 to -10 from the proximal end of the oviduct, which produced statistically-indistinguishable DNA content distributions. 2N DNA content was established from non-mitotic somatic cells of the vulva and uterus in the same animal and from sets of daughter chromosomes of anaphase germ nuclei, and was verified using 4N nuclei (metaphase figures and pachytene nuclei). To obtain the haploid equivalent, the total fluorescence from each germ cell nucleus was divided by one half of the 2N value obtained from the somatic cells. Every nucleus was measured from the distal tip to the first cell diameter within four cell diameters of the transition zone (to avoid meiotic S) as described previously [31].

### Germ cell and somatic cell radiosensitivity assays

Radiation-induced germ cell apoptosis was analyzed as per [28]. Worms were synchronized at 25°C and irradiated at the L4 stage. Germ cell corpses were scored in the distal pachytene region of one gonad arm of wild-type worms, and in both distal and proximal regions of one gonad arm of *glp-1(ar202)*. Radiation-induced somatic phenotypes were assessed by vulval morphology in adults derived from 120Gy-irradiated late-stage embryos (at 4h after egg laying). Vulval phenotypes are scored as wild-type or abnormal including protruding vulva (Pvl), vulvaless (Vul), ruptured vulva (Rup) and uncoordinated (Unc) using Nomarksi microscopy. Phenotype percentages were derived from animals surviving until adulthood. To examine meiotic chromosomes, L4 hermaphrodites were subjected to 120Gy, and after 18h DAPI-stained oocytes at diakinesis were evaluated under a Zeiss fluorescence equipped with epifluorescence filters.

### Antibody staining

For anti-PhosphoTyr15-CDK-1 immunostaining, gonads were dissected from adult worms into M9 [32], fixed 5 min in -20°C methanol, blocked 30 min in 0.5% BSA in PBST (0.05% Tween-20 in PBS), and then incubated at 4°C overnight in a 1:250 dilution of anti-P-Tyr15-CDK-1 antiserum (Calbiochem) in PBST. Gonads were incubated for 2h at room temperature with rhodamine-conjugated goat anti-rabbit antiserum (Invitrogen) diluted 1:250 in 0.5% BSA in PBST. Images were collected from a Zeiss Imager Z1 with Apotome (Carl Zeiss Inc.) using an AxioCamMRm digital camera and Zeiss AxioVision and NIH ImageJ software.

### Worm longevity studies

Assays were performed at 25°C. Synchronized L4-stage worms, timed to egg laying, were placed on seeded plates on day one. Adults were transferred from progeny onto fresh plates every other day until reproduction ceased. Data, derived from animals scored daily as dead or alive, is plotted as Kaplan-Meier survival curves using Graphpad Prism.

### Cell culture

CUTLL-1 cells, a gift from Dr. Adolfo Ferrando (Institute for Cancer Genetics, Columbia University)[26], were cultured in RPMI 1640 media supplemented with 20% fetal bovine serum, 100 U⁄mL penicillin G, and 100 μg⁄mL streptomycin at 37°C in a humidified atmosphere under 5% CO_2_. CUTLL-1 cell cycle distribution was analyzed as per Rodriguez et al. [33].

### RAD51 shRNA

CUTLL-1 cells were infected with GIPZ Lentiviral particles expressing human *RAD51* shRNA or non-silencing shRNA (Open Biosystems Inc. RAD51 clone ID V2LHS_171184). Stable cell lines were selected by addition of 1 μg⁄ml puromycin and GFP expression. Efficiency of *RAD51* knockdown was measured by quantitative PCR as above. Human *RAD51* expression level was normalized to human TATA-binding protein (*TBP*) expression (Open Biosystems, Inc. RAD51 assay ID is Hs-00153418 and TBP assay ID is Hs-433769-0711011).

### XRCC4 shRNA

shRNA sequences were predicted by the Designer of Small Interfering RNAs (DSIR) software (http://biodev.extra.cea.fr/DSIR/DSIR.html). Multiple shRNA sequences were tested in order to achieve high knockdown efficiency. The shRNA constructs were cloned into the pHAGE-puro vector and transfected into 293T cells with delta 8.9 and pMDG vectors to produce lentivirus. CUTLL-1 cells were infected with unconcentrated virus overnight at 37°C and puromycin selected the next day. Efficiency of *XRCC4* knockdown measured by quantitative PCR was 65% compared to empty vector-treated CUTLL-1 cells. Level of human *XRCC4* expression was normalized to human TATA-binding protein (*TBP*) expression (Open Biosystems Inc. XRCC4 assay ID is Hs-01104868).

### Clonogenic survival assay

Cells (0.5x10^6^⁄ml complete media) were subjected to escalating radiation doses. At 1h post irradiation, cells were added into Methylcellulose Medium (Stemcell Technologies) working solution containing 20% fetal bovine serum according to manufacturer's instructions. The cell suspension was seeded onto 35 mm dishes in triplicate and after 11–14 days, surviving colonies, defined as a minimum of 50 cells, were counted using a stereoscopic microscope (Nikon TMS). Surviving fraction (SF) was calculated as number of colonies formed⁄number of cells seeded x plating efficiency. Radiation dose survival curves were fitted to the LQ standard model [34] using GraphPad Prism 6. D_0_ (the dose required to reduce the fraction of surviving cells to 37% of its previous value) and D_q_ (a threshold dose below which there is no effect) were calculated as Nomiya T described [34]. To test radiation-drug combination effect, cells were treated with Mirin (provided by the Organic Synthesis Core Facility, MSK) for 1h preceding irradiation, followed by a 12-day drug-free clonogenic assay.

### Notch-driven tumor irradiation studies

6–8 week old non-obese diabetic⁄severe combined immunodeficient (NOD-SCID) female mice were purchased from Taconic Farms Inc. Mice were housed at the MSK animal core facility. Xenografted tumors were generated in murine right flanks using 5x10^6^ CUTLL-1 cells infected with GIPZ shRNA non-silencing lentiviral particles or cells infected with GIPZ human *RAD-51* shRNA lentiviral particles, selected as described above. At 100–150 mm^3^, tumors were irradiated using a Philips MG-324 X-ray unit at 117.5 cGy⁄min (50 cm source to skin distance). Tumor volumes were measured 2x per week for at least 15 weeks. Euthanasia is performed by exposing mice to 100% carbon dioxide in a cage or euthanasia chamber as recommended in The American Veterinary Medical Association (AVMA) Guidelines for the Euthanasia of Animals (2013, pp. 26, M1.6).

This study was carried out as recommended in the Guide for the Care and Use of Laboratory Animals of the National Institutes of Health. The protocol was approved by the Institutional Animal Care and Use Committee of Memorial Sloan-Kettering Cancer Center (IACUC protocol 92–10–038). All procedures performed comply with provisions of the Animal Welfare Act. Memorial Sloan-Kettering Cancer Center’s animal care and use program is administered by the Research Animal Resource Center (RARC). The program has been fully accredited by the Association of Assessment and Accreditation of Animal Care, International (AAALAC) since 1967, is registered with the USDA, and has an approved assurance on file with the Office of Laboratory Animal Welfare, NIH (OLAW).

## Statistical Analysis

Statistical significance was determined by a two-tailed Student t-test using GraphPad Prism software (GraphPad, San Diego, CA, USA). Results are presented as mean ± standard error. The *P* value in clonogenic survival of CUTLL-1 cells was calculated from the confidence interval as defined by Altman and Bland [35].

## Results and Discussion

### Profiling ionizing radiation impact on germline tumors in GLP-1⁄Notch *gf* mutants

**Fig 1:**
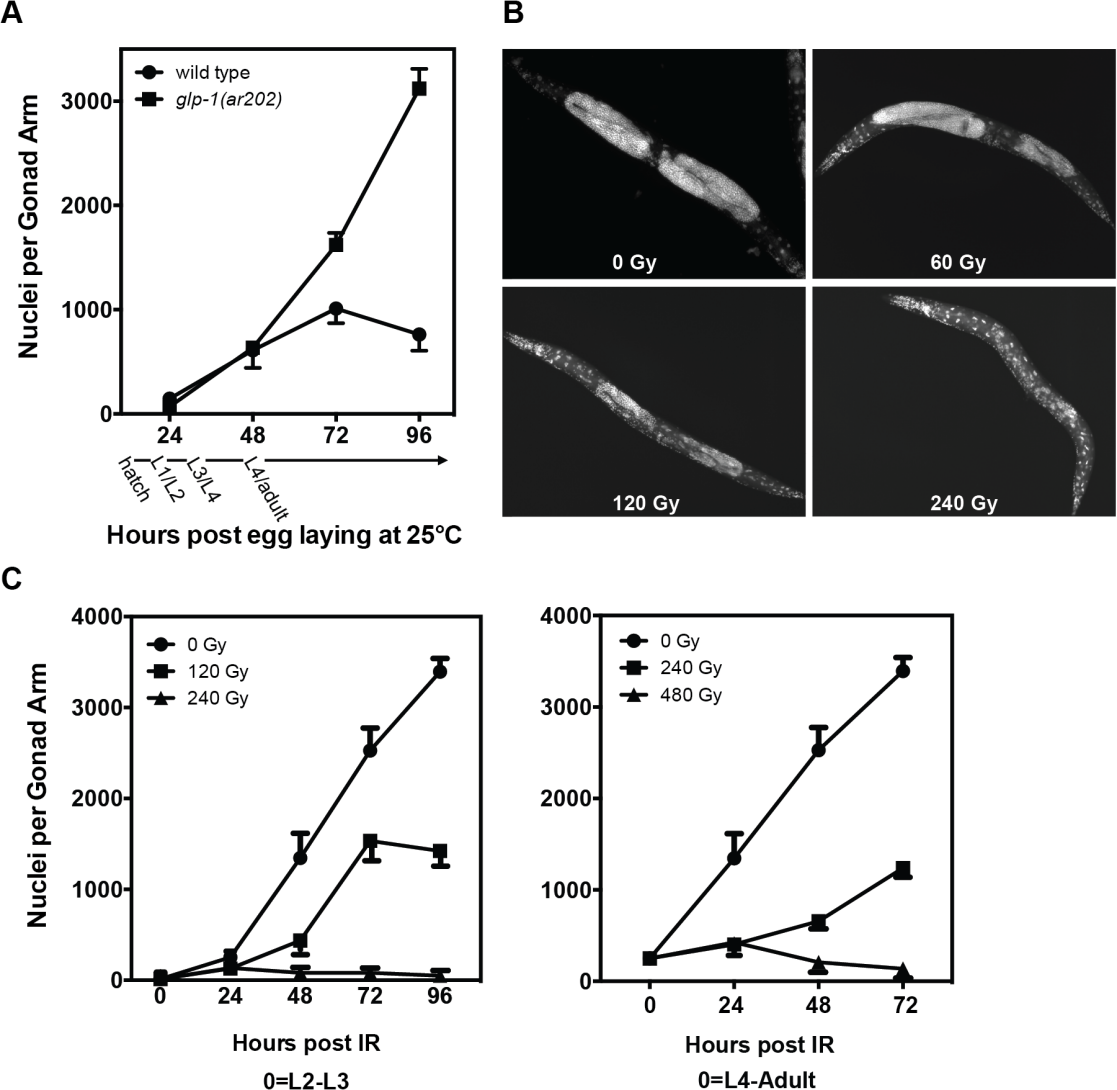
Response of *C*. *elegans* germline tumors to ionizing radiation. (A) Time course of germ cell accumulation in wild-type and *glp-1(ar202)* hermaphrodites. Worms were stained with DAPI at the indicated times after egg laying and imaged (20x magnification). Data (mean±s.e.m) represent number of germ nuclei⁄gonad in a minimum of 10 gonad arms. (B) Representative images of germline tumors in adult *glp-1(ar202)* post radiation. Worms were irradiated at the L2-L3 stage (30h after egg laying) and DAPI stained at 40h post irradiation. (C) Stage sensitivity of germline tumors to ionizing radiation. *glp-1(ar202)* were irradiated at the L2-L3 or late L4 stage, and quantified as in (A). All experiments were performed at 25°C as described in Methods.

We first defined *glp-1(ar202)* radiosensitivity. At 15°C, the permissive temperature, GSC number is highly regulated through 4 larval stages into adulthood [15]. At 25°C, however, incessant germline proliferation occurs such that by 96h post egg laying (late L4⁄early adult stage), average number of nuclei⁄gonad is four-fold higher than wild-type worms (3,121 vs. 762⁄gonad, *p*<0.001; Fig 1A), associated with 50% shortened lifespan (*p*<0.001 vs. wild-type). For further details, please see Fig 3D. Exposing *ar202* mutants to ionizing radiation at 30h after egg-laying (late L2⁄early L3 stage) results in dose-dependent germline lethality with tumor abrogation at 240Gy (Fig 1B, quantified in Fig 1C, left), sustained for the lifespan of the worm. When irradiated in late L4⁄young adult stage (50h after egg-laying) at 240Gy, germline tumor cells were more radioresistant (Fig 1C, right), with significant dose-dependent germline reduction at 72h, but without tumor eradication up to 480Gy [252±36 cells⁄pre-irradiated L4 gonad arm (n = 10) vs. 137±11 cells⁄gonad arm after 480Gy (n = 9), *p*<0.01]. Thus the range of 120-480Gy appears appropriate for defining elements of *C*. *elegans* DNA damage response (DDR) and mechanisms of radiation lethality for this Notch-driven *glp-1(ar202)* tumor model.

### Ionizing radiation induces cell cycle arrest in G2⁄M and non-apoptotic cell death in *ar202*

**Fig 2:**
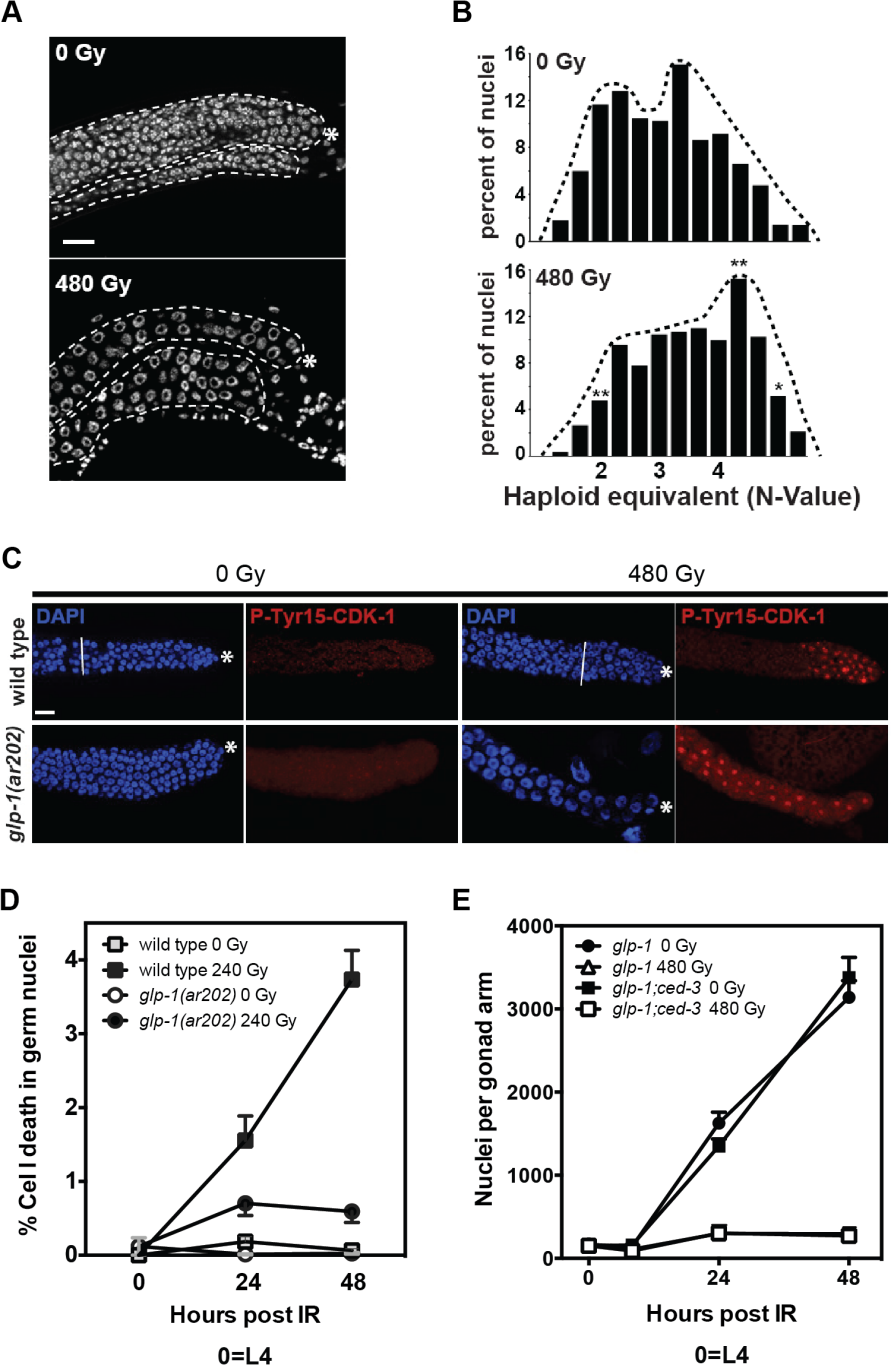
Tumor cells in *glp-1(ar202)* arrest in G2 phase following radiation exposure, and are apoptosis resistant. (A) Worms were irradiated at L4 stage and after 12h stained with DAPI. Representative germline tumors are outlined. (B) Relative nuclear DNA content of distal germ nuclei in unirradiated (0Gy) or irradiated (480Gy) worms. A total of 207 nuclei from 5 unirradiated worms, and 147 nuclei from 5 irradiated worms were scored. (*p<0.05; **p<0.01 relative to non-irradiated control). (C) Wild-type and *glp-1(ar202)* unirradiated or irradiated germline are stained with anti-phospho-Tyr15-CDK-1 antiserum (red) and DAPI (blue) as in Methods. White bar indicates border of proliferative zone. Asterisk indicates position of distal end. Scale bar is 20 μm. (D) Comparison of radiation-induced germ cell apoptosis in wild type and *glp(ar202)*. Wild type and mutant worms were synchronized at 25°C and irradiated with 240Gy at the L4 stage. Germline apoptosis was scored in one gonad loop per worm. Incidence of germ cell death was quantified by dividing number of apoptotic germ cells by total germ cells. Data (mean±s.e.m) are from 10–12 worms⁄group. (E) Inactivation of apoptosis does not alter *ar202* response to radiation. *glp-1(ar202)* and *glp-1(ar202);ced-3(n717)* double mutant worms were irradiated at the L4 stage. Data (mean±s.e.m) are from 9–12 worms⁄group. Note the line of “*glp-1* 480Gy” is hidden behind the line of “*glp-1;ced-3* 480Gy”.

A hallmark of the eukaryotic DDR is cell cycle arrest, which facilitates coordinated deployment of multistage DNA repair systems or evolution of apoptotic death [36–38]. Here we show that when treated in L4 with a maximally-effective dose of 480Gy, *glp-1(ar202)* germline tumor cells exhibit rapid increase in nuclear size (Fig 2A, 8h), with average volume increasing from 63.4±1.0 μm^3^ to 145.3±2.5 μm^3^ (*p*<0.01, S1 Fig), associated with significant shift toward higher DNA content (Fig 2B), consistent with previous observations on cell cycle blockade in irradiated *C*. *elegans* [39], suggesting cell cycle arrest occurring at late S-G2**⁄**M phase. Further, phospho-Tyr15-CDK-1, an established biomarker of G2⁄M arrest in response to DNA damage [40], while not detected in germ cells of unirradiated wild-type or *glp-1(ar202)* gonads, was found in virtually all proliferative germ nuclei in both wild-type and *glp-1(ar202)* worms exposed to 480Gy. In wild-type worms, P-Tyr15-CDK-1 was present at 8h post-irradiation in 91% of proliferative zone nuclei, ending abruptly as germ cells entered meiotic prophase (n = 318 nuclei⁄6 gonads, Fig 2C). Consistent with all germ cells in *glp-1(ar202)* as phenotypically similar to wild-type proliferative zone cells, P-Tyr15-CDK-1 was observed in 93% of post-irradiation tumor nuclei. Subsequent to G2⁄M cell cycle arrest, significant *ar202* germ cell loss occurred, detected at 12h post 480Gy (young adult stage) [265±36 in pre-irradiated L4 (n = 21) vs. 162±11 (n = 22), *p<*0.01].

We and others reported that wild-type worms show dose-dependent germline apoptosis after irradiation, confined to cells in meiotic prophase just distal to the gonad arm bend [28,39,41]. To determine if radiation-induced apoptotic cell death contributes to germ cell loss in *ar202*, we irradiated worms and examined germ cell apoptosis at 24h and 48h post radiation. However, there was little *ar202* germ cell apoptosis after 240Gy (Fig 2D) or 480Gy (not shown). Since caspase gene *ced-3* is required for radiation-induced germline apoptosis [42], *ced-3* was inactivated by 2 approaches in *glp-1(ar202)*, either by generating a *ced-3(n717);glp-1(ar202)* double mutant or by RNAi, and germ cell number was scored after irradiation. Inactivation of caspase-mediated cell death by either approach did not alter *ar202* radiation response (Fig 2E, S2 Fig), indicating radiation-induced germline loss in *ar202* is non-apoptotic.

### *glp-1(ar202)* germline tumor cells engage homology-directed repair (HDR) for radioprotection

**Fig 3:**
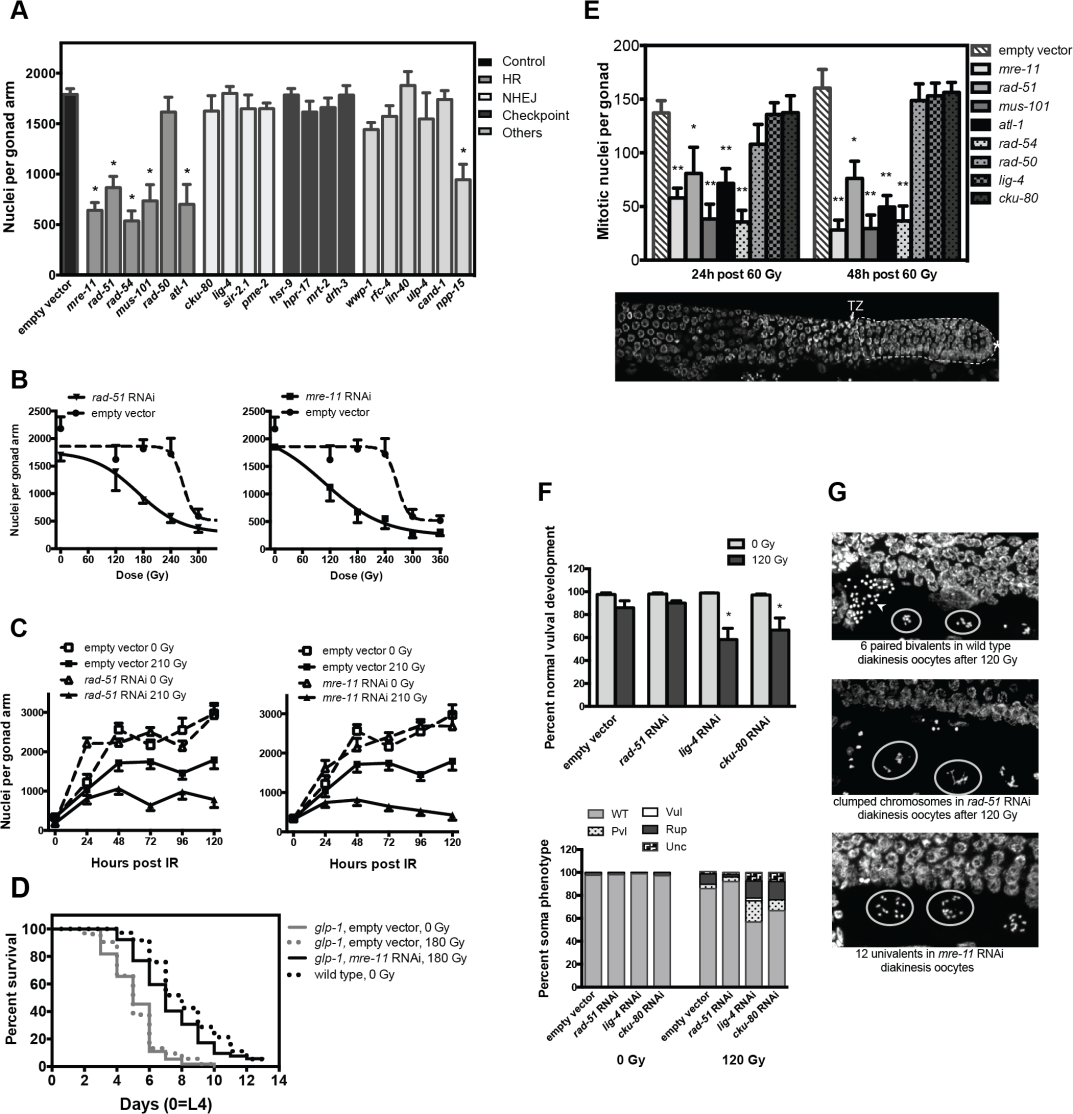
Radiation sensitivity of germline tumor after RNAi. (A) Knockdown of HDR ortholog genes radiosensitizes *ar202* tumors. L4 *ar202* worms were irradiated at 210Gy, an ineffective dose in this strain as shown in Fig 3B, and germline nuclei were quantitated at 72h post irradiation. Genes knocked down are classified according to their function in DNA damage repair pathways. Asterisks indicate significantly increased radiation sensitivity compared with empty vector control group, *p<0.01. (B) Inactivation of *rad-51* (left) or *mre-11* (right) enhances radiosensitivity of germ cells. L4 *ar202* worms were irradiated and germline nuclei counted 72h post radiation. Data (mean±s.e.m) are from 5–8 worms⁄group. Note the empty vector data set is the same in left and right panel. (C) Time course of germ cell accumulation after 210Gy combined with *rad-51* RNAi (*left*) or *mre-11* RNAi (*right*). Data (mean±s.e.m) are from 7–12 worms⁄group. Note the empty vector data set is the same in left and right panel. (D) *mre-11* inactivation extends survival of *glp-1(ar202)* after 180Gy treatment of L4 larvae. Survival assays were performed at 25°C. Data are from one representative of 3 experiments scoring ≥50 animals per group. (E) Knockdown of HDR genes radiosensitizes mitotic germ cells in distal gonad of wild-type worms. L4 stage-worms were irradiated with 60Gy and mitotic germ cells were quantified as in Fig 1A. Mitotic germ cells reside between the distal end of the gonad (indicated by bold asterisk in bottom panel) and the transition zone [10], which characteristically contains crescent-shaped nuclei (arrow). *p<0.05 and **p<0.01 vs. empty vector control. (F) Knockdown of NHEJ genes results in vulval abnormalities post irradiation. Phenotypes were evaluated 120h post 120Gy using 75–85 worms⁄group. Somatic developmental phenotypes were quantified as wild-type vulva (WT), protruding vulva (Pvl), vulvaless (Vul), ruptured vulva (Rup) and uncoordinated (Unc). *p<0.05. (G) Knockdown of HDR genes in wild-type worms results in highly-abnormal oocyte chromosome morphology post irradiation. Chromosome morphology was quantified in the two oocytes (circled) closest to the spermatheca (arrow in right upper panel) at 18h post irradiation. Quantification of these data is included in Table 2.

An alternative death pathway might entail reproductive (mitotic) cell death, an outcome of failure of cycling cells to adequately repair DNA DSBs, usually by coordinate activation of NHEJ and HDR [43]. To explore mechanisms of DSB repair in *glp-1(ar202)*, we employed RNAi knockdown of the conserved DDR repair machinery. Quantitative PCR confirmed RNAi knockdown efficiency (S3 Fig). Table 1 and Fig 3A summarize impact of DDR gene silencing. RNAi depletion of 5⁄6 HDR genes *(mre-11*, *rad-51*, *rad-54*, *mus-101*, *atl-1*, but not *rad-50*), and the *npp-15* ortholog of human NUP133, a mammalian nuclear pore component [44], conferred radiosensitivity. Unlike other HDR genes, *rad-50* knockdown in mutant *glp-1(ar202)* does not enhance radiosensitivity in mitotic germline tumors, although *rad-50* gene expression was reduced after RNAi by 81±8% in *ar202* (S3 Fig), indicating that *C*. *elegans* RAD-50 may not play a role in radiation-induced DSB repair in mitotic germ cells. This result is consistent with findings from Villeneuve and co-workers that showed RAD-50 is required for loading RAD-51 onto radiation-induced DSBs in meiotic but not mitotic germ cells [45].

**Table 1:** RNAi of DDR orthologs detects genes that enhance *ar202* tumor radiosensitivity.

	Gene	Human Ortholog	Function	Enhancement of radiosensitivity	Experiments	N*
1	*mre-11*	*MRE11A*	HR	Yes	18	526
2	*rad-51*	*RAD51*	HR	Yes	11	430
3	*rad-54*	*RAD54L*	HR	Yes	3	48
4	*mus-101*	*TOPBP1*	HR	Yes	4	53
5	ra*d-50*	*RAD50*	HR	No	3	50
6	*atl-1*	*ATR*	HR	Yes	4	59
7	*cku-80*	*XRCC5 (Ku80)*	NHEJ	No	3	70
8	*lig-4*	*LIG4*	NHEJ	No	4	63
9	*sir-2*.*1*	*SIRT1*	NHEJ	No	2	42
10	*pme-2*	*PAPR2*	NHEJ⁄DNA damage checkpoint	No	3	58
11	*hsr-9*	*TP53BP1*	DNA damage checkpoint	No	3	48
12	*hpr-17*	*RAD17*	DNA damage checkpoint	No	2	30
13	*mrt-2*	*RAD1*	DNA damage checkpoint	No	3	48
14	*drh-3*	*IFIH1*	DNA damage checkpoint	No	2	32
15	*wwp-1*	*WWP1 and WWP2*	Ubiquitin protein ligase	No	2	31
16	*rfc-4*	*RFC4*	DNA replication	No	2	38
17	*lin-40*	*MTA1*	Histone deacetylase complex	No	2	40
18	*ulp-4*	*SENP7NUP133*	ubiquitin-like protease	No	2	32
19	*cand-1*	*CAND1⁄TIP120A*	Encodes TATA-binding protein	No	2	35
20	*npp-15*	*NUP133*	Nuclear pore complex protein	Yes	3	48

N* represents number of animals examined

Detailed analysis of impact of inactivating *rad-51* and *mre-11* revealed significantly-increased sensitivity of *glp-1(ar202)* germ cells between 60-300Gy, reducing 50% tumor control dose from 266 to 168Gy with *rad-51* RNAi (Fig 3B, left; *p*<0.01) and to 105Gy for *mre-11* RNAi (Fig 3B, right; *p*<0.01). Differences in tumor response were detectable at 24h after 210Gy (Fig 3C; *p*<0.01), and at 120h *rad-51*-inactivated worms displayed 74% reduced germ cell number (2,973 vs. 782 GSCs⁄gonad), while *mre-11* inactivation nearly eradicated tumor. Furthermore, *mre-11* RNAi treatment was associated with extension of *ar202* lifespan post-irradiation, comparable to that of wild-type unirradiated worms (Fig 3D). In contrast to HDR genes, silencing genes of canonical NHEJ (*cku-80* and *lig-4*), cell cycle, DNA damage checkpoint, DNA replication and chromatin remodeling had no impact on *ar202* germline tumor radiosensitivity (Fig 3A and Table 1). RNAi conferred similar radiation responses in germ cells in the distal region of wild-type worms, enhancing radiosensitivity at 60Gy, an ineffective dose in N2 worms (not shown), upon knockdown of HDR (*mre-11*, *rad-51*, *rad-54*, *mus-101* and *atl-1;* Fig 3E *)*, but not NHEJ (*lig-4* and *cku-80*) genes.

To address whether *ar202* germline tumors express NHEJ genes, we employed the temperature-sensitive germ cell-deficient mutant *glp-4(bn2)*[46]. S1 Table shows that when *glp-4(bn2)* animals are grown at the permissive temperature, and therefore contain a germ line, they express key NHEJ genes *lig-4* and *cku-80*, as well as HDR genes *mus-101*, *rad-51* and *atl-1*, at much higher levels than animals grown at the restrictive temperature, which lack a germ line. Gene expression levels in somatic tissue and germ line could also be affected by culturing animals at the different temperatures, although this is unlikely in our study. We conclude, therefore that NHEJ genes are, in fact, enriched in the germ line, while post-mitotic somatic cells in adult worms express minimal amounts. Consistent with these data, we recently reported mitotically-active cells of murine small intestinal crypts aggressively repair radiation DNA damage, while post-mitotic villus cells do not [23].

To obtain functional evidence that RNAi feeding adequately inactivated respective NHEJ DSB repair genes, we examined consequence of inactivating NHEJ genes on somatic development in irradiated wild-type worms. For these studies, N2 embryos grown in *lig-4* RNAi plates were collected at 4h post egg laying, a time preceding vulval development, and irradiated with 120Gy. At 96h after 120Gy, minimal overall damage was detected in N2 worms even with *rad-51* silencing, while *lig-4* or *cku-80* knockdown-worms displayed abnormal vulval development (Fig 3F, upper panel, *p*<0.01 for *lig-4; p*<0.05 for *cku-80*), with increased penetrance of somatic defects (lower panel) [47,48]. Taken together, our results suggest that failure of germline tumors to use NHEJ to repair radiation-induced DSBs results from lack of engagement of NHEJ repair machinery, rather than lack of availability of NHEJ repair genes in the germline.

Investigation of germline chromosomal aberrations produced results consistent with this finding as only HDR gene inactivation yielded post-radiation germline chromosomal aberrations (Fig 3G). Diakinesis oocytes in control worms usually display the normal number of six bivalents (visualized by DAPI, corresponding to six paired homologs attached by chiasmata) at 18h after 120Gy. While neither *cku-80* nor *lig-4* RNAi impacted this post-radiation pattern (Table 2), *rad-51* RNAi yielded high frequency of clustered chromosomes. Loss-of-function *mre-11* displayed, in addition to clumping, twelve univalents within oocytes [49] (Fig 3G and Table 2). Altogether these studies suggest an exclusive role for HDR in the reparative response of Notch-responsive proliferating germ cells to ionizing radiation. Furthermore, the NHEJ apparatus appears available in the germline but apparently not engaged for DSB repair, suggesting NHEJ is actively suppressed in germ cells, consistent with prior reports [47,50].

**Table 2:** Knockdown of HDR genes results in abnormal morphology in *C*. *elegans* oocytes.

RNAi	Dose Gy	Average chromosomes per oocyte	Normal oocytes (%)	Clustered oocytes (%)	Total oocytes examined
**control**	0	5.8	107 (100)	0	107
	120	5.7	86 (100)	0	86
***mre-11***	0	10.5	63 (100)	0	63
	120	7.1	23 (43.4)	30 (56.6)	53
***rad-51***	0	5.5	49 (96.0)	2 (3.9)	51
	120	5.5	31(54.4)	26 (45.6)	57
***lig-4***	0	5.7	67 (100)	0	67
	120	5.6	40 (100)	0	40
***cku-80***	0	5.8	80 (97.6)	2 (2.4)	82
	120	5.6	24 (96.0)	1 (4.0)	25

15–25 animals were examined per group at each dose

### Inactivation of HDR radiosensitizes human Notch-driven cancer

**Fig 4:**
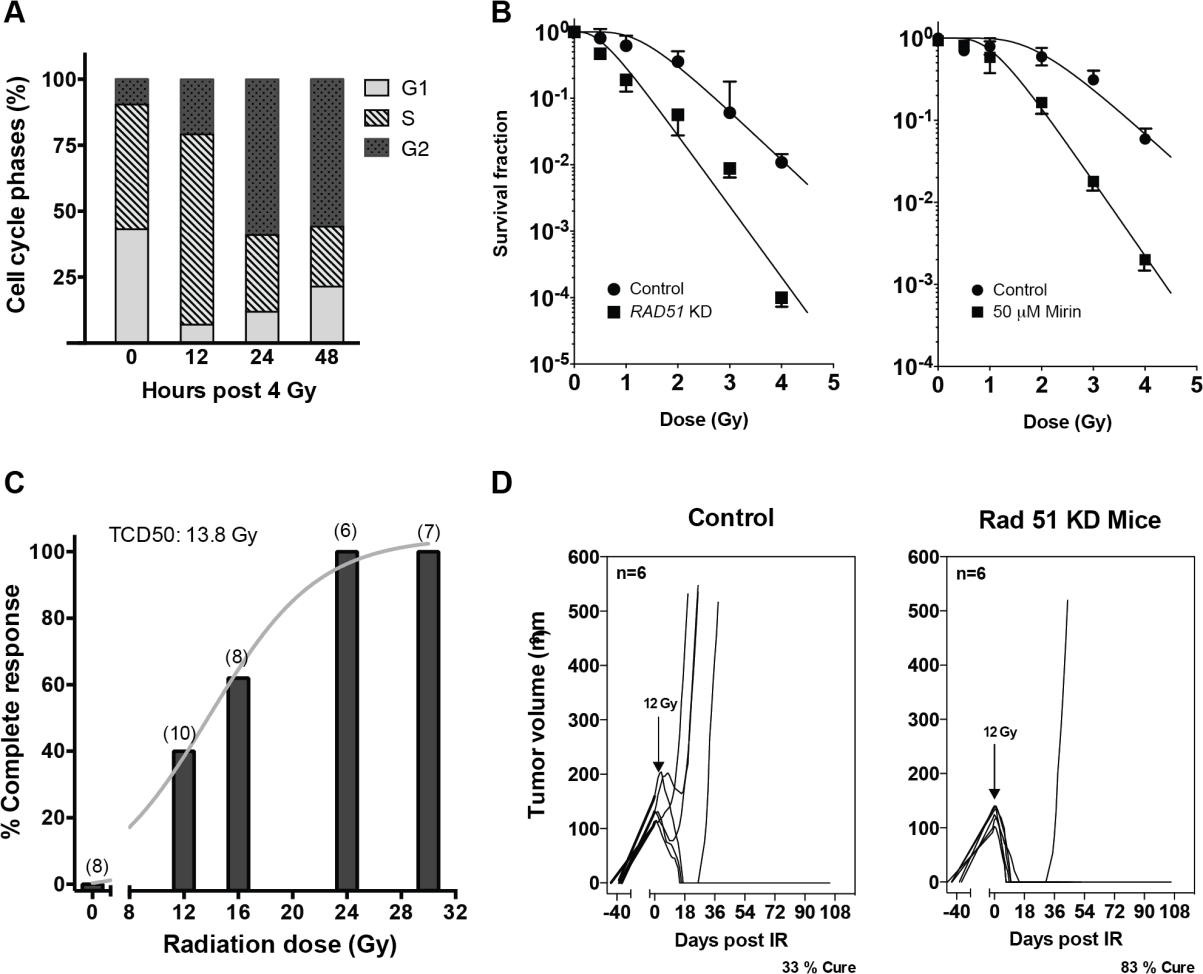
HDR inactivation radiosensitizes human Notch-driven cancer. (A) Cell cycle distribution of unirradiated and 4Gy-treated CUTLL-1 cells. DNA content was assessed by propidium iodide incorporation and FACS analysis. (B) Targeting *RAD51* or inhibiting the Mre11-Rad50-Nbs1 complex radiosensitizes CUTLL-1 cells. Clonogenic survival was performed in CUTLL-1 cells expressing human *RAD51* shRNA or non-silencing control shRNA (left), or in cells treated with or without 50 μM Mirin (right). Surviving colonies (**>**50 cells**)** were scored at 11–14 days post irradiation. Data represent three independent experiments for each assay. (C) Complete CUTLL-1 tumor response after single dose radiotherapy. CUTLL-1 chloromas (100–150 mm^3^) in flanks of NOD-SCID female mice were irradiated, and tumor volumes were measured using calipers 2x weekly for 3 months. Complete response was defined as lack of measurable tumor. Parentheses denote number of mice⁄group. The curve was fit to data by nonlinear regression analysis using the Prism Sigmoidal Curve Fit program. (D) *RAD51* inactivation radiosensitizes Notch-driven tumors. NOD-SCID female mice harboring *RAD51* shRNA-expressing CUTLL-1 xenografts (KD *RAD51*) or non-silenced control CUTLL-1 tumors (control) were treated with 12Gy and tumor size measured as in (C).

Aberrant Notch activation occurs in diverse human cancers, such as in breast cancer and T-ALL [2,5], although the role of Notch in human cancer remains enigmatic and therapeutic gain has not yet been realized by targeting a Notch phenotype [51]. To test whether inhibiting HDR radiosensitizes Notch-driven human malignancy, we employed the T-cell lymphoblastic lymphoma cell line CUTLL-1 [26], which harbors a t(7;9) translocation producing hyperactive NOTCH1, similar to *glp-1(ar202)*. Irradiated CUTLL-1 cells display fewer cells in G1⁄S relative to G2 with G2 phase cells increasing from 9.2% at baseline to >55% at 24h after 4Gy, which persists for 48h (Fig 4A). To silence *RAD51*, CUTLL-1 cells, infected with human *RAD51*GIPZ lentiviral shRNA, were puromycin selected, leading to 33% stable *RAD51* reduction (S4 Fig). *RAD51* shRNA-expressing CUTLL-1 cells displayed significantly-reduced colony formation with D_0_ of the radiation dose-response curve shifting from 0.59 to 0.40 (*p<*0.001), and minimal impact on D_q_ (Fig 4B, left). A similar result was obtained by administering the small molecule MRE11⁄HDR inhibitor Mirin [52]. Irradiated-CUTLL-1 cells, pre-treated for 1h with 50 μM Mirin, a dose that does not affect cell survival (S5 Fig), followed by a 12-day drug-free clonogenic assay, exhibited radiosensitization comparable to genetic *RAD51* knockdown (D_0_ decreasing from 0.77 to 0.47 with Mirin; Fig 4B right). In contrast, knockdown of the critical NHEJ repair gene *XRCC4* was not radiosensitizing (S6 Fig).

To test whether targeting HDR would enhance in vivo-radiosensitivity in Notch-driven cancer, *RAD51* shRNA-expressing CUTLL-1 cells, grown as chloromas in the flanks of immunodeficient (NOD-SCID) mice, were irradiated at 100–150 mm^3^. Initial studies established the 50% tumor control dose (TCD_50_), a standard readout of radiotherapy effectiveness [53], as 13.8Gy for CUTLL-1 tumors (Fig 4C). A 12Gy-dose was selected to evaluate impact of RAD51 inactivation. *RAD51*-shRNA-expressing CUTLL-1 xenografts responded to 12Gy more robustly than non-silenced control CUTLL-1 tumors (*p<*0.001), with all *RAD51* shRNA-expressing CUTLL-1 tumors showing complete responses by 10 days. Further, over 15 weeks, 83% of *RAD51* shRNA-expressing CUTLL-1 tumors achieved autopsy-confirmed cure, while only 33% of CUTLL-1 tumors expressing non-silencing shRNA achieved cure (Fig 4D), equivalent to a 1.5-fold dose-modifying factor for radiosensitization based on HDR inactivation.

Tumor radiosensitization is of fundamental importance to radiation oncologic research, although successes have been modest, as tumor-specific DDR phenotypes tractable for pharmacologic intervention remain poorly defined. Here, we characterize a radiation phenotype in a NOTCH-driven *C*. *elegans* stem cell tumor that predicts pharmacologic and genetic outcome of human NOTCH-driven tumor radiosensitization. These studies provide a basis for clinical strategies for improved NOTCH-directed cancer therapy using agents currently under development that target HDR.

## Supporting Information

S1 Fig Irradiation of *glp-1(ar202)* increases average size of germ cell nuclei. 480Gy-treated *glp-1(ar202)* germline tumor cells display increased average volume in both distal and proximal tumorous germline (n = 883 nuclei from 20 gonads at 0Gy, and n = 710 nuclei from 20 gonads at 480Gy). Asterisks indicate *p<*0.01.
(TIF)

S2 Fig Inactivation of caspase-mediated cell death did not alter *ar202* radiation response. (A) Inactivation of *ced-3* using RNAi does not alter *ar202* response to radiation. Worms were irradiated at the L4 stage and germ nuclei counted in one gonad arm at 72h post radiation. Data (mean±s.e.m) represent number of germ nuclei per gonad from ≥10 worms per group. (B) As an RNAi assay control, radiation-induced germ cell apoptosis was measured in wild-type worms with *ced-3* RNAi. Worms were irradiated at the L4 stage and apoptotic cells were scored at 30h post radiation. Data (mean±s.e.m) are from 8–11 worms per group.
(TIF)

S3 Fig Efficiency of RNAi knockdown in *glp-1(ar202)*. Extent of RNAi-induced knockdown in *glp-1(ar202)* was estimated for 8 genes in parallel with germline proliferation assays. Gene expression levels were analyzed by qPCR as in Methods. All samples were run in triplicate and standard deviations were <1.5%. Error bars indicate s.e.m from ≥3 independent experiments.
(TIF)

S4 Fig Inactivation of *RAD51* in CUTLL-1 cells. (A) After puromycin selection, human *RAD51* GIPZ lentiviral-transduced CUTLL-1 cells display high-level GFP expression (200x magnification). (B) Level of *RAD51* gene knockdown analyzed by qPCR. Control represents CUTLL-1 cells infected by non-silencing lentiviral shRNA. Error bar indicates s.e.m. collated from 3 independent experiments.
(TIF)

S5 Fig Mirin enhances radiosensitivity of CUTLL-1 cells. CUTLL-1 cells were treated with 0–100 μM Mirin for 1h before irradiation. Number of cell colonies (mean±s.e.m.) were scored on day 12.
(TIF)

S6 Fig Knockdown of *XRCC4* in CUTLL-1 cells is not radiosensitizing. (A) After puromycin selection, human *XRCC4* lentiviral-transduced CUTLL-1 cells display high-level tdTomato expression (200x magnification). (B) Level of *XRCC4* gene knockdown analyzed by qPCR. Control represents empty vector-treated CUTLL-1 cells. Error bars indicate s.e.m. collated from 3 independent experiments. (C) Clonogenic survival in CUTLL-1 cells expressing human *XRCC4* shRNA. Surviving colonies **(>**50 cells) were scored at 11–14 days post irradiation.
(TIF)

S1 Table Distribution of DNA repair gene expression in somatic tissue and germ line. (DOCX)
