# Correction: Targeting Homologous Recombination in Notch-Driven *C*. *elegans* Stem Cell and Human Tumors

**DOI:** 10.1371/journal.pone.0141673

**Published:** 2016-01-22

**Authors:** Xinzhu Deng, David Michaelson, Jason Tchieu, Jin Cheng, Diana Rothenstein, Regina Feldman, Sang-gyu Lee, John Fuller, Adriana Haimovitz-Friedman, Lorenz Studer, Simon Powell, Zvi Fuks, E. Jane Albert Hubbard, Richard Kolesnick

The Funding statement is incorrect. The correct Funding statement should be: This work was supported by grants from the National Institutes of Health (Grant No. R01 GM 061706 to EJAH) and from the National Cancer Institute (Grant No. P30 CA008748).
