# Supplementary figures and images for: Targeting Homologous Recombination in Notch-Driven C. elegans Stem Cell and Human Tumors
Source: PLoS One. 2015 Jun 29;10(6):e0127862. doi: 10.1371/journal.pone.0127862 (PMC4485896; doi:10.1371/journal.pone.0127862)

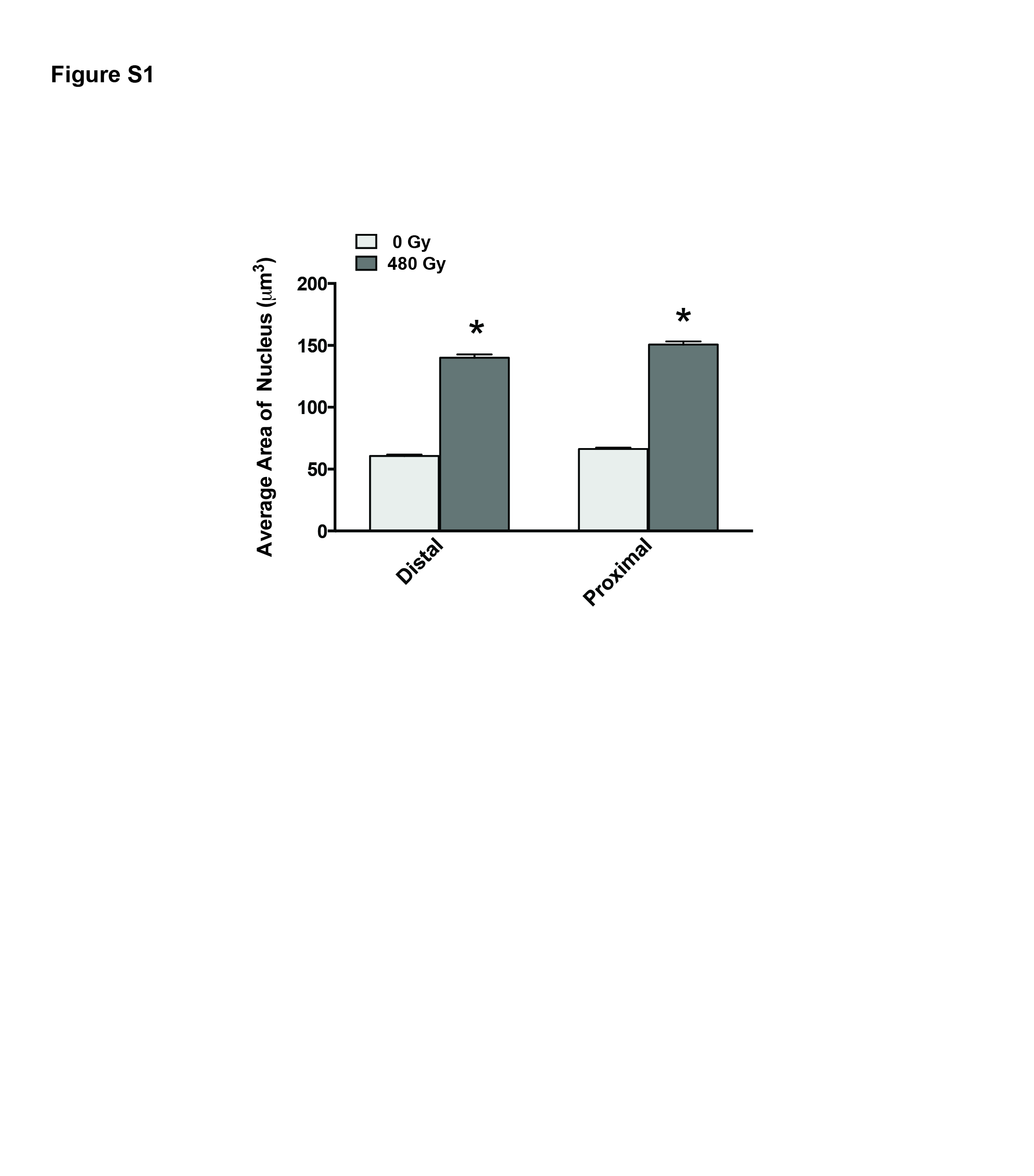

Supplement: S1 Fig — 480Gy-treated glp-1(ar202) germline tumor cells display increased average volume in both distal and proximal tumorous germline (n = 883 nuclei from 20 gonads at 0Gy, and n = 710 nuclei from 20 gonads at 480Gy). Asterisks indicate p<0.01. (TIF) [file pone.0127862.s001.tif]

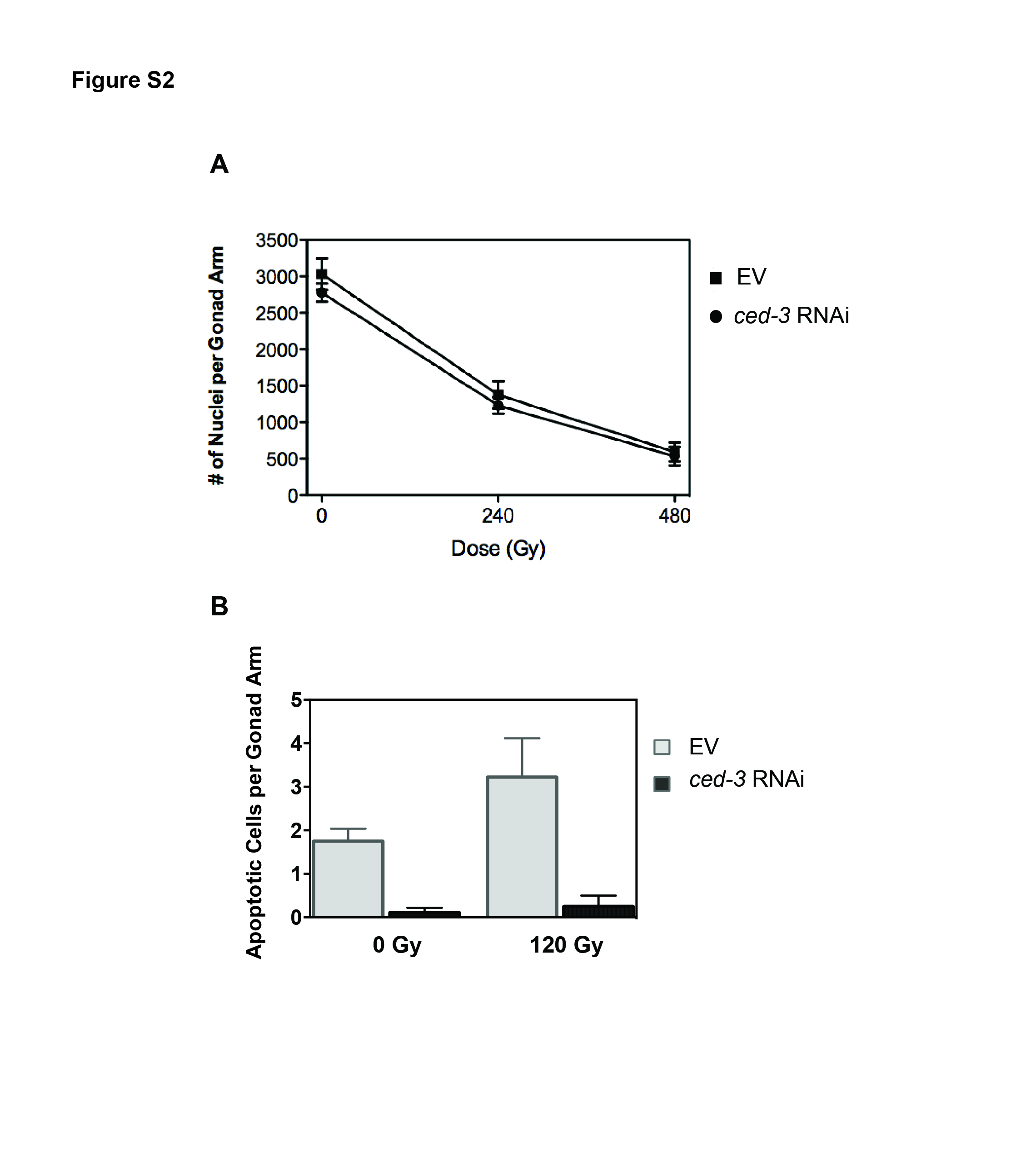

Supplement: S2 Fig — (A) Inactivation of ced-3 using RNAi does not alter ar202 response to radiation. Worms were irradiated at the L4 stage and germ nuclei counted in one gonad arm at 72h post radiation. Data (mean±s.e.m) represent number of germ nuclei per gonad from ≥10 worms per group. (B) As an RNAi assay control, radiation-induced germ cell apoptosis was measured in wild-type worms with ced-3 RNAi. Worms were irradiated at the L4 stage and apoptotic cells were scored at 30h post radiation. Data (mean±s.e.m) are from 8–11 worms per group. (TIF) [file pone.0127862.s002.tif]

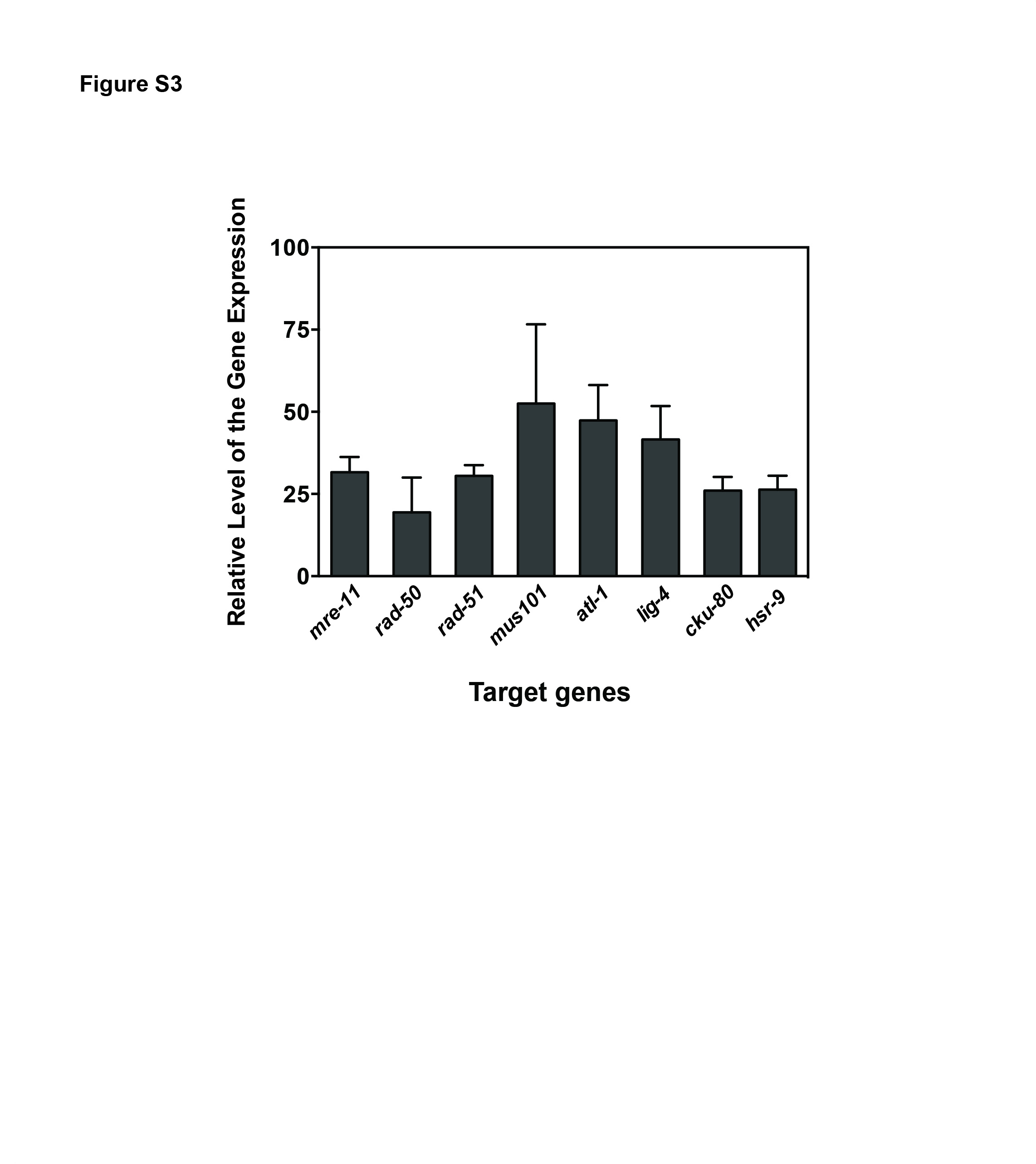

Supplement: S3 Fig — Extent of RNAi-induced knockdown in glp-1(ar202) was estimated for 8 genes in parallel with germline proliferation assays. Gene expression levels were analyzed by qPCR as in Methods. All samples were run in triplicate and standard deviations were <1.5%. Error bars indicate s.e.m from ≥3 independent experiments. (TIF) [file pone.0127862.s003.tif]

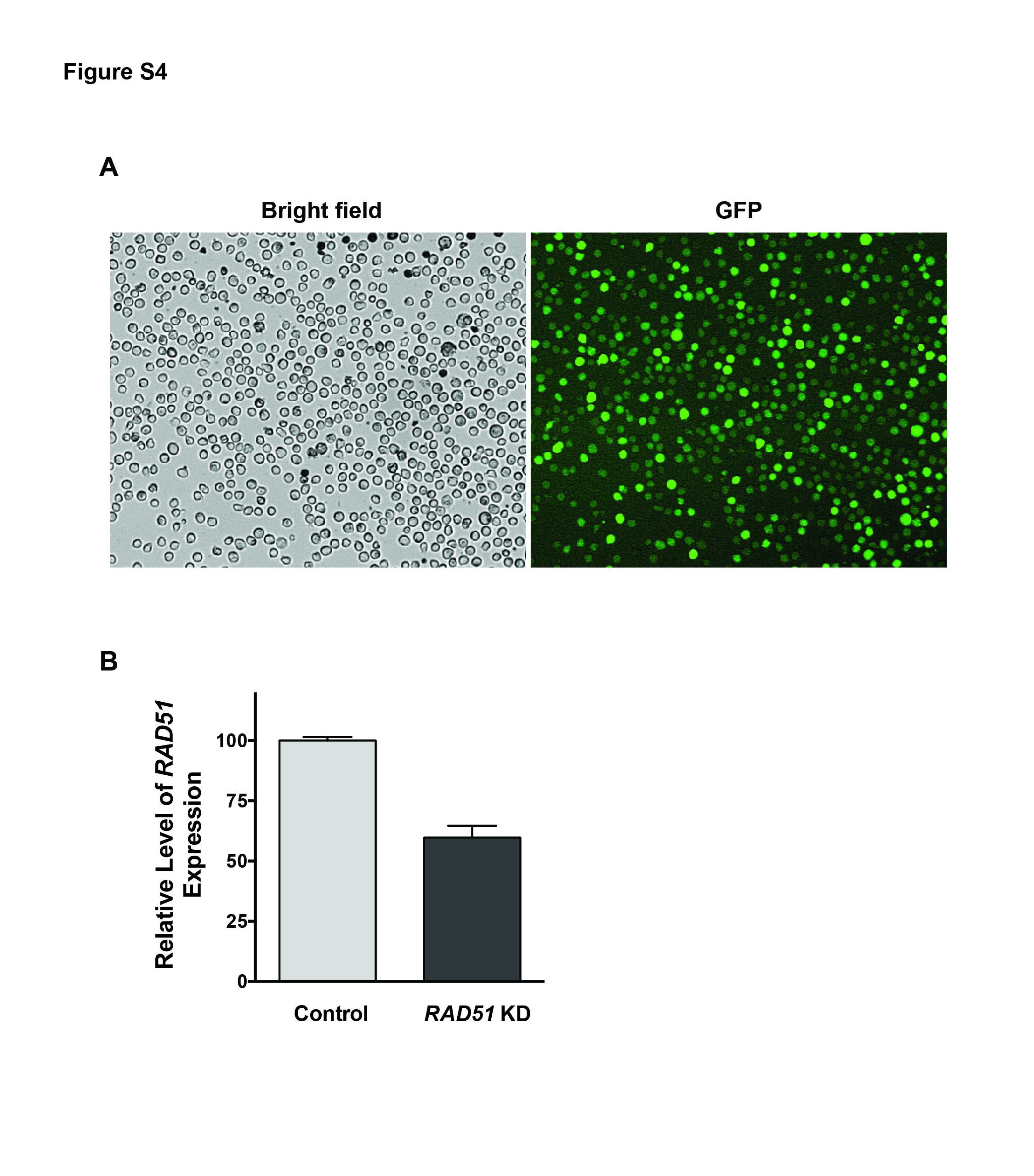

Supplement: S4 Fig — (A) After puromycin selection, human RAD51 GIPZ lentiviral-transduced CUTLL-1 cells display high-level GFP expression (200x magnification). (B) Level of RAD51 gene knockdown analyzed by qPCR. Control represents CUTLL-1 cells infected by non-silencing lentiviral shRNA. Error bar indicates s.e.m. collated from 3 independent experiments. (TIF) [file pone.0127862.s004.tif]

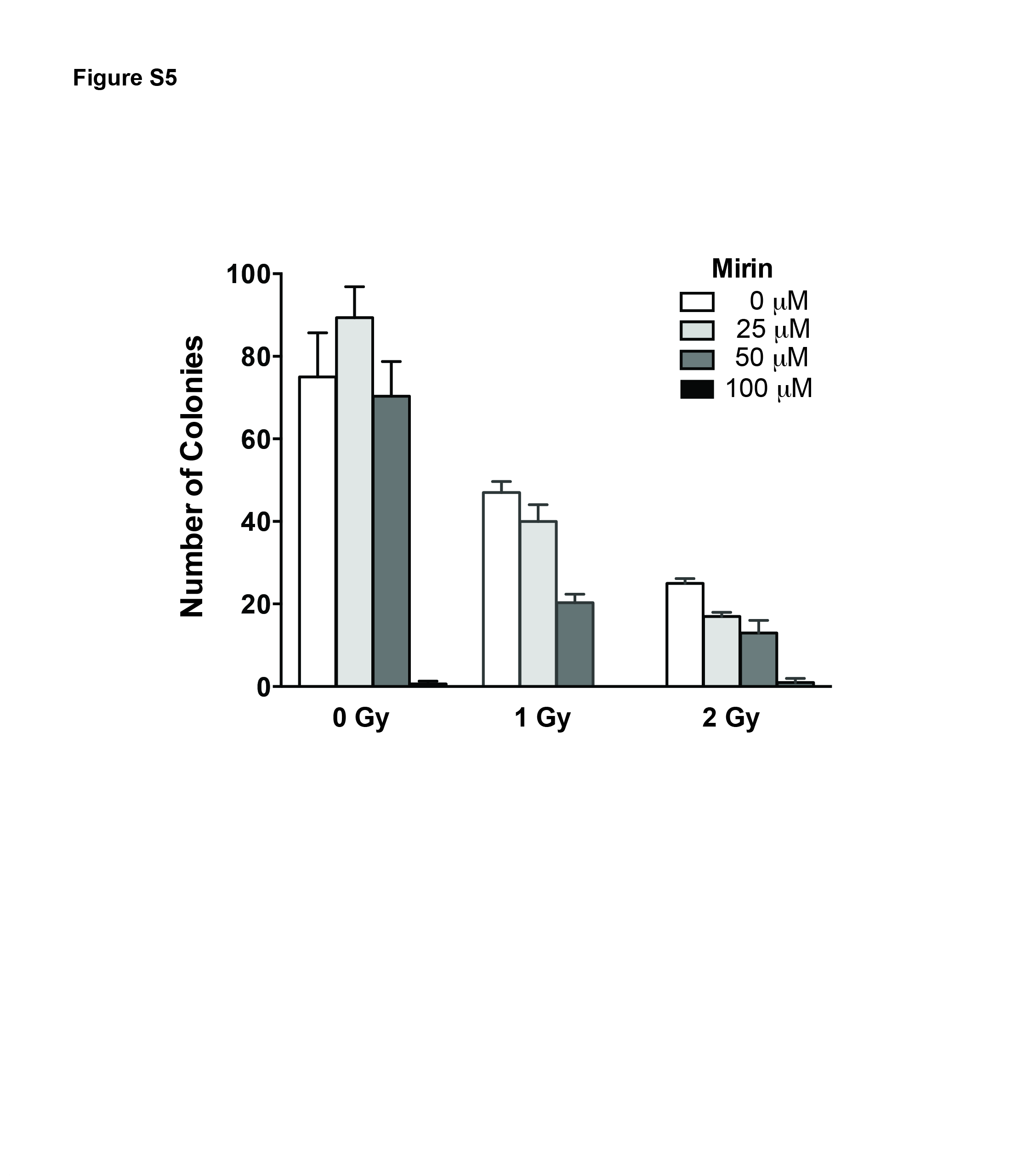

Supplement: S5 Fig — CUTLL-1 cells were treated with 0–100 μM Mirin for 1h before irradiation. Number of cell colonies (mean±s.e.m.) were scored on day 12. (TIF) [file pone.0127862.s005.tif]

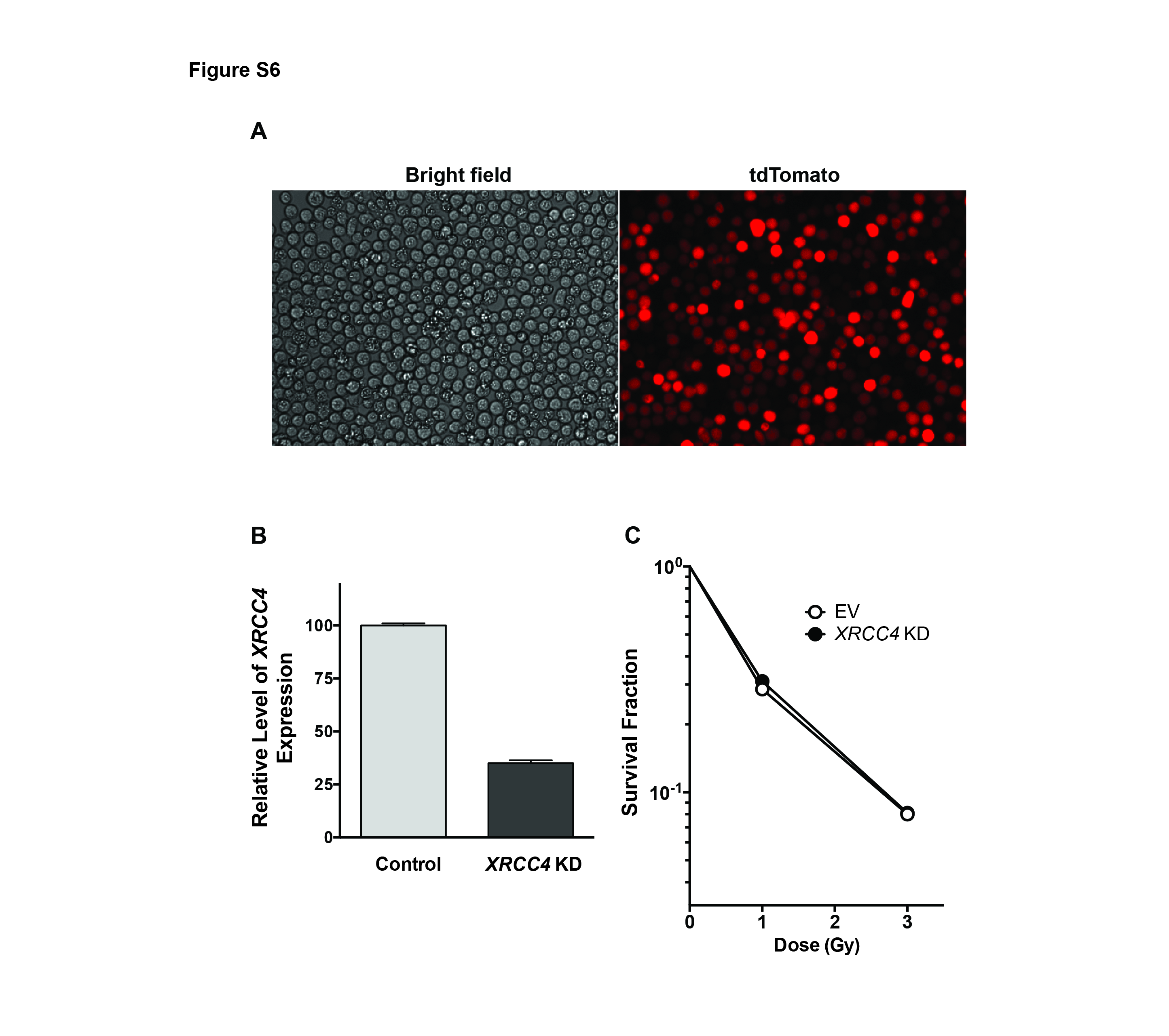

Supplement: S6 Fig — (A) After puromycin selection, human XRCC4 lentiviral-transduced CUTLL-1 cells display high-level tdTomato expression (200x magnification). (B) Level of XRCC4 gene knockdown analyzed by qPCR. Control represents empty vector-treated CUTLL-1 cells. Error bars indicate s.e.m. collated from 3 independent experiments. (C) Clonogenic survival in CUTLL-1 cells expressing human XRCC4 shRNA. Surviving colonies (>50 cells) were scored at 11–14 days post irradiation. (TIF) [file pone.0127862.s006.tif]
