# Supplementary material for: Targeting Homologous Recombination in Notch-Driven C. elegans Stem Cell and Human Tumors
Source: PLoS One. 2015 Jun 29;10(6):e0127862. doi: 10.1371/journal.pone.0127862 (PMC4485896; doi:10.1371/journal.pone.0127862)
Supplement: S1 Table — (DOCX) [file pone.0127862.s007.docx]

**Table S1**

**Distribution of DNA repair gene expression**

**in somatic tissue and germ line**

| **Genes** | ***glp-4(bn2)***  **at 15º**  **soma + germline** | ***glp-4(bn2)***  **at 25º**  **soma** | **Germline**  **expression**  **(%)** |
| --- | --- | --- | --- |
| *lig-4* | 100 | 14.3 | 85.7 |
| *cku-80* | 100 | 3.0 | 97.0 |
| *mus101* | 100 | 4.8 | 95.2 |
| *rad-51* | 100 | 2.5 | 97.5 |
| *atl-1* | 100 | 5.2 | 94.8 |

A mixed stage *glp-4(bn2)* population was cultured at 15ºC to measure gene expression in whole worm tissue, and at 25ºC to measure only somatic tissue gene expression by qPCR. Relative gene expression in the germ line was obtained by subtracting the level of somatic tissue from whole worm expression.
